# Ursodeoxycholic acid and bezafibrate were useful for steroid-refractory, immune-related hepatitis: a case report

**DOI:** 10.1186/s13256-020-02541-3

**Published:** 2020-11-26

**Authors:** Sachiyo Onishi, Masahiro Tajika, Hideaki Bando, Yuki Matsubara, Waki Hosoda, Kei Muro, Yasumasa Niwa

**Affiliations:** 1 Department of Endoscopy, Aichi Cancer Center Hospital, 1-1 Kanokoden, Chikusa-ku, Nagoya, 464-8681 Japan; 2 Department of Clinical Oncology, Aichi Cancer Center Hospital, 1-1 Kanokoden, Chikusa-ku, Nagoya, 464-8681 Japan; 3 Department of Pathology and Molecular Diagnostics, Aichi Cancer Center Hospital, 1-1 Kanokoden, Chikusa-ku, Nagoya, 464-8681 Japan

**Keywords:** Nivolumab, Hepatic immune-related adverse events, Ursodeoxycholic acid, Bezafibrate

## Abstract

**Background:**

Immune checkpoint inhibitors have shown clinically significant antitumor efficacy and have been approved for the treatment of various kinds of advanced malignancies. On the other hand, these immunotherapies show unique adverse events, termed “immune-related adverse events,” which are distinctly associated with conventional cytotoxic chemotherapy. Hepatotoxicity is recognized as an immune-related adverse event; prompt treatment with corticosteroids is recommended. However, some cases are refractory to steroids. Here, we report the first case (to our knowledge) of steroid-refractory immune-related hepatitis that was successfully treated with ursodeoxycholic acid and bezafibrate.

**Case presentation:**

A 68-year-old Asian man, came to our hospital for the treatment of malignant melanoma involving the gingiva and presenting with multiple lymph node and bone metastases was administered nivolumab as a first-line treatment. Two months into treatment, the patient developed diarrhea as a result of immune-related colitis; the colitis was treated successfully with prednisolone 60 mg/ day, resulting in improvement in the patient’s symptoms. However, when steroids were being tapered, acute elevation of liver enzymes was observed. Autoimmune hepatitis was suspected as an immune-related adverse event, and treatment with intravenous prednisolone 60 mg/ day was reinitiated. However, restoration of the steroid treatment failed to improve the patient’s liver enzymes. On the basis of histological findings from liver biopsy and exclusion of other etiologies such as viral infection and other drug-induced hepatitis, steroid-refractory hepatic immune-related adverse event was deemed the most likely cause of the patient’s acute hepatitis. In general, mycophenolate mofetil or tacrolimus is known to provide benefits in cases of steroid-refractory hepatitis. We therefore decided to add oral ursodeoxycholic acid and bezafibrate in consideration of the patient’s background of repeated aspiration pneumonia. Administration of this regimen resulted in an improvement in liver function, which remained normal even after tapering of prednisolone.

**Conclusions:**

Ursodeoxycholic acid and bezafibrate may be useful for treatment of steroid-refractory immune-related adverse event hepatitis.

## Introduction

Tumors resist immune attack by inducing tolerance among tumor-specific T cells and by expressing ligands that engage inhibitory receptors and dampen T-cell functions within the tumor microenvironment [1]. Antibody blockade of these immune checkpoints can significantly enhance antitumor immunity [2]. Major immune checkpoint inhibitors include anticytotoxic T-lymphocyte-associated antigen 4 (CTLA-4) antibody and anti–programmed cell death 1 (anti–PD-1) [3–5] antibody. PD-1 is a key immune checkpoint receptor expressed by activated T cells, and this membrane protein mediates immunosuppression. Inhibition of the interaction between PD-1 and its ligand, programmed death ligand 1 (PD-L1), can enhance T-cell responses and mediate antitumor activity [6–8]. Nivolumab, an anti–PD-1 antibody, is currently approved for the treatment of malignant melanoma, non–small cell lung cancer, renal cell carcinoma, Hodgkin lymphoma, head and neck cancer, gastric cancer, malignant pleural mesothelioma, microsatellite instability–high colon cancer, and esophageal cancer. Immune checkpoint inhibitors such as nivolumab have completely different mechanisms from conventional antitumor and molecularly targeted drugs, expanding clinical options for cancer treatment. On the other hand, immune checkpoint inhibitors are associated with specific immune-related adverse events (irAEs) that are distinct in both mechanism and management from the adverse effects commonly associated with chemotherapy. irAEs such as pneumonitis, hypothyroidism, arthralgia, and vitiligo are more common with anti–PD-1 antibody [9]. The incidence of the all-grade hepatic irAEs in cases being treated with anti–PD-1 antibodies accounts for 1–3% of adverse events in these patients [10, 11]. In general, corticosteroids are recommended as first-line treatment for hepatic irAEs. However, in steroid-refractory cases, mycophenolate mofetil (MMF) or tacrolimus may provide benefit [10]. Treatment of steroid-refractory hepatic irAE is controversial. However, prolongation of treatment for irAE may lead to interruption of treatment for the primary disease. We report a case of steroid-refractory hepatic irAE after administration of nivolumab. The irAE was confirmed by histological findings, and improvement was obtained by treatment with ursodeoxycholic acid (UDCA) and bezafibrate in combination with corticosteroids.

## Case presentation

**Fig. 1:**
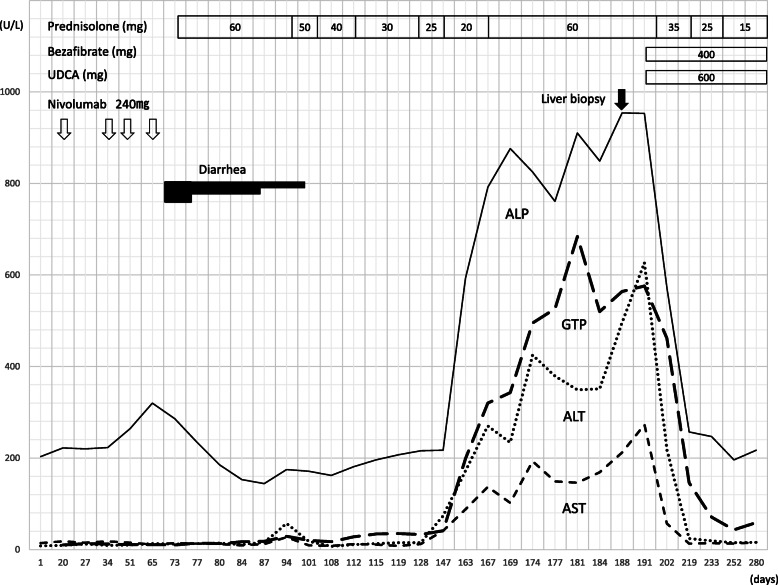
Chronological changes of the patient’s liver function tests. The first nivolumab dose was administered on day 1. The patient’s serum AST, ALT, g-GTP, and ALP levels did not decrease appreciably after the amount of prednisolone was increased. We then initiated administration of UDCA 600 mg/day and bezafibrate 400 mg/day. After the start of UDCA and bezafibrate administration, the serum levels of AST, ALT, g-GTP, and ALP decreased, even with a restart of prednisolone tapering. *ALP* Alkaline phosphatase, *ALT* Alanine aminotransferase, *AST* Aspartate aminotransferase, *g-GTP* Gamma-glutamyl transpeptidase, *UDCA* Ursodeoxycholic acid

**Fig. 2:**
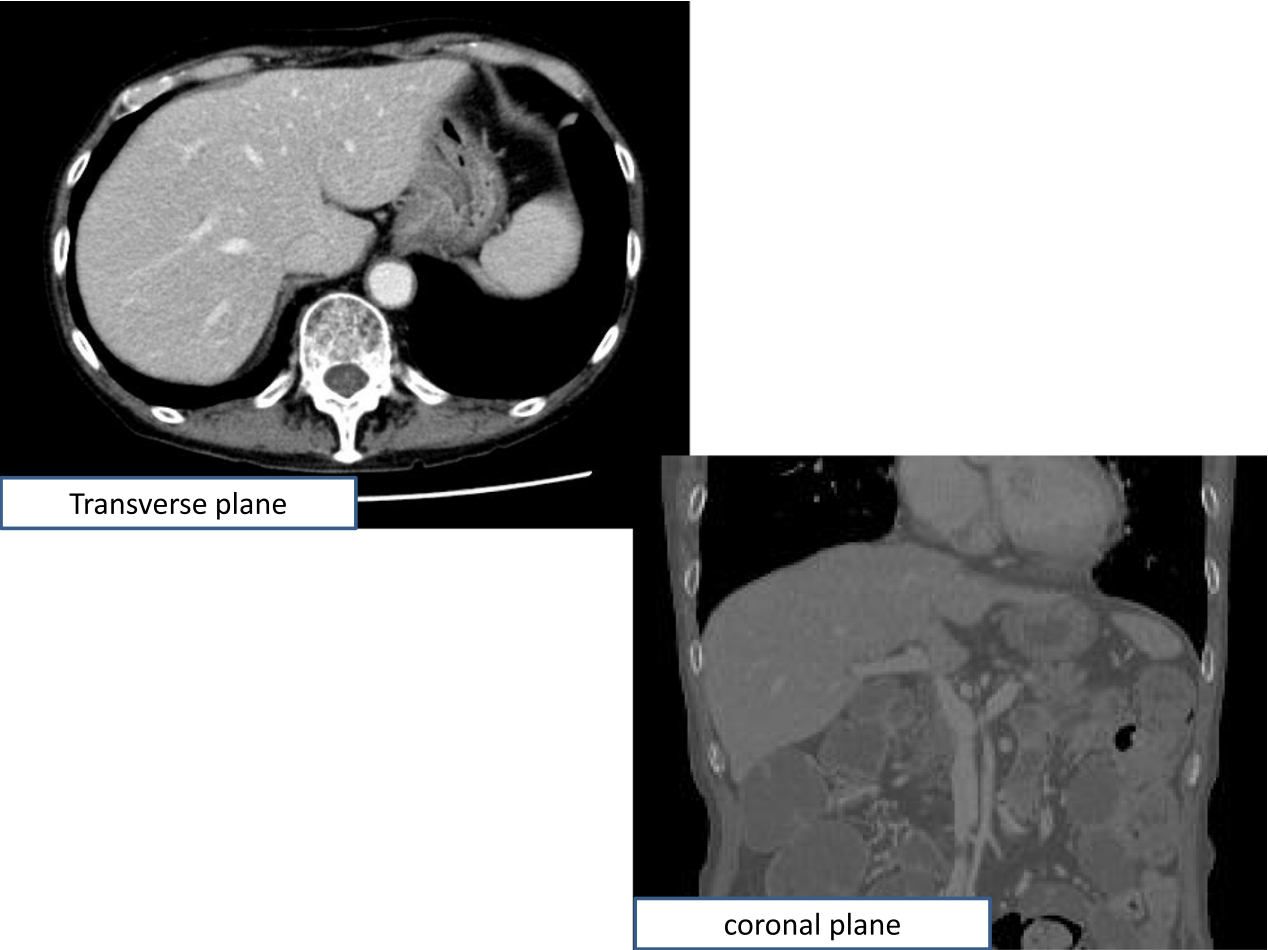
Abdominal computed tomography at the time of liver dysfunction. There were no occupational lesions in the hepatics and no dilation of either the intrahepatic or extrahepatic bile ducts

Our patient was a 68-year-old Asian man. His past medical, social, environmental, family, and employment histories were unremarkable. He had a history of surgery for duodenal ulcer when he was 20 years old. He had been both smoking and drinking for almost 50 years. Upon admission to our institute, the patient was conscious and well oriented to time, place, and person. His general examination revealed that he was thin. His blood pressure was 157/88 mmHg, and his pulse rate was 82 beats/minute. His systemic examination did not reveal any abnormality. He had multiple lymph node metastases and bone metastases of malignant melanoma involving the gingiva. The patient was administered nivolumab as a first-line treatment. Two months after the first treatment with four courses of nivolumab (240 mg every 2 weeks), the patient was hospitalized with severe diarrhea (> 10 episodes/day) without abdominal pain, despite not having taken any medications that might be expected to cause diarrhea. Routine stool cultures, tests for *Clostridium difficile* toxin, and tests for cytomegalovirus (CMV) infection yielded negative results. Colonoscopy showed mucosal ulceration throughout the entire colon, and histopathologic analysis showed focal active colitis with crypt destruction and inflammatory cell infiltration in the crypt epithelium, conditions most consistent with nivolumab-associated enterocolitis. Therefore, our diagnosis was grade 3 diarrhea due to irAE enterocolitis. The patient was not administered further nivolumab. To manage the irAE enterocolitis, dosing with intravenous prednisolone at 1 mg/kg/day (60 mg/day) was started. The patient’s diarrhea improved rapidly after the initiation of prednisolone treatment. We subsequently switched to oral prednisolone and eventually tapered the dose to 20 mg/day. However, laboratory tests revealed a sudden elevation of liver enzymes halfway through tapering (Fig. 1), although the patient did not exhibit any abdominal pain or abdominal tenderness. The interval between the initiation of corticosteroid treatment and the onset of liver dysfunction was 142 days. Whole-body computed tomography and abdominal ultrasonography showed only a fatty liver; no sign of biliary tract disease was apparent (Fig. 2). Laboratory testing for liver disease was performed. Dyslipidemia was found to be slightly higher than baseline values. The patient had no jaundice or renal dysfunction (total bilirubin 0.3 mg/dl, blood urea nitrogen 18 mg/dl, creatinine 0.83 mg/dl). Abnormalities in albumin (2.9 mg/dl), C-reactive protein (6.79/μl), white blood cell count (11,740/μl), red blood cell count (419 × 10^4^/μl), and platelet count (42.6 × 10^4^/μl) were also present. The patient had a negative test result for antinuclear antibody and anti–smooth muscle antibody. Active viral hepatitis A, B, C, and E were excluded. Although the patient had a previous positive test result for CMV immunoglobulin G (IgG), subsequent immunoglobulin M (IgM) testing was equivocal. The finding of testing for Epstein-Barr virus (EBV) anti–virus capsid antigen (anti-VCA) IgM was negative, whereas the findings for anti-EBV nuclear antigen and EBV anti-VCA IgG were positive. The clinical findings were consistent with grade 3 hepatic irAE secondary to nivolumab.

**Fig. 3:**
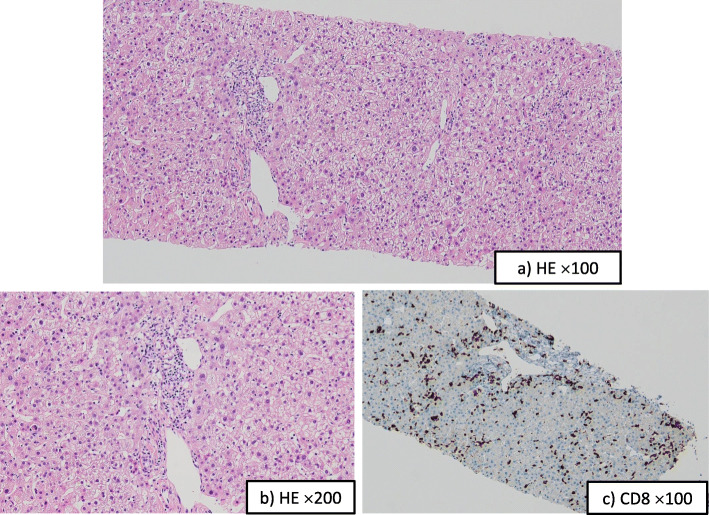
Histological findings of the liver. **a** and **b** Photomicrographs of a representative hematoxylin and eosin (H&E) section from the liver biopsy (**a**: H&E, original magnification × 100; **b**: H&E, original magnification × 200). Moderate inflammation and focal necrosis were observed. The liver parenchyma was mainly damaged with moderate infiltration of lymphocytes. **c** Photomicrograph shows CD8 immunohistochemistry of a f3:4 representative section from the liver biopsy. Most of the infiltrating lymphocytes were positive for CD8 staining (brown)

We decided to reinitiate administration of intravenous prednisolone at 1 mg/kg/day (60 mg/day). However, the patient’s transaminase levels subsequently rose rather than fell (Fig. 1). Further increasing the dose of prednisolone was not considered feasible, given the repeated occurrence of aspiration pneumonia under the influence of underlying malignant melanoma of the gingiva. To clarify the etiology of the patient’s liver dysfunction, a liver biopsy was performed. Histological examination of the specimen showed inflammatory cell infiltrates, mostly comprising CD8-positive lymphocytes. Plasma cell infiltration was not conspicuous, suggesting autoimmune hepatitis was not involved. No evidence of biliary disorder (such as shedding of biliary epithelium) was seen, suggesting a finding of hepatocellular hepatitis (Fig. 3). By exclusion of other etiologies and because of occurrence of the event despite previously having been on corticosteroid treatment, steroid-refractory hepatic irAE was deemed to be the most likely cause of the patient’s abnormal liver biochemistry results. Therefore, we considered additional immunosuppressive therapies such as MMF or tacrolimus. However, we were hesitant to implement these treatments in consideration of this patient’s background of repeated aspiration pneumonia. The liver biopsy did not show peribiliary inflammation, but the laboratory data showed a marked elevation of serum alkaline phosphatase (ALP) and γ-glutamyltranspeptidase. Given the possible presence of bile congestion with dyslipidemia, we added UDCA 600 mg and bezafibrate 400 mg to the patient’s medication, a combination that has been reported to improve bile congestion, as in the treatment of primary biliary cirrhosis (PBC) [12]. These medications were administered with the patient’s informed consent. After the start of this new oral pharmacotherapy, the patient’s serum levels of liver enzymes improved rapidly (Fig. 1). The prednisolone dose was subsequently tapered to 35 mg/day, and the patient was finally discharged. Despite this reduction in prednisolone dose, his transaminase levels normalized after an additional 35 days of oral prednisolone administration. Prednisolone was still being tapered without recurrence of diarrhea or elevation of transaminases in serum. Because of the observed severe adverse events, nivolumab was not restarted. The patient’s general condition was not good, and he accepted palliative care and was transferred to a hospital in order to receive palliative care about 1 year after the first visit.

## Discussion

We report a case of steroid-refractory hepatic irAE after administration of nivolumab, and improvement was obtained by treatment with UDCA and bezafibrate in combination with corticosteroids. Nivolumab is a monoclonal antibody targeting PD-1. Agents against PD-1 may be associated with serious irAEs, which arise as a consequence of impaired self-tolerance from loss of T-cell inhibition [13]. Hepatic irAEs occur less frequently than those in other systems. Hepatic irAEs most often become clinically evident between 8 and 12 weeks after initiation of therapy but may occur as late as 1 year after the first dose [14]. The mechanism of these adverse events is not fully understood. Hepatic irAEs have been classified into three types: hepatocellular, cholestatic, and mixed. Cholestatic or mixed-type liver injury has been seen more frequently in patients receiving anti-PD-1 and/or anti-PD-L1 antibodies than in those receiving anti-CTLA-4 antibodies [15]. For any type of hepatic irAE, prompt treatment with corticosteroids is recommended. Literature suggests that the median time to resolution is approximately 8 weeks [16]. In cases of steroid-refractory immune-related hepatitis, MMF or tacrolimus may prove beneficial [16]. However, steroids and other immunosuppressants may have a limited utility in severe injury with biliary involvement [17]. The resolution of hepatic irAE may require prolonged treatment, and long-term administration of immunosuppressants can result in significant complications. The histopathology of hepatic irAE is thought to be distinct from the characteristic features of classical autoimmune hepatitis, which typically is marked by plasma cell infiltration, rosette formation, and interface hepatitis. A previous study reported that hepatic irAEs were characterized predominantly by lobular hepatitis with infiltration by CD3^+^ or CD8^+^ lymphocytes, but not by CD20+ lymphocytes [18]. In our case, liver biopsy demonstrated moderate hepatitis, but fibrosis was not seen with hematoxylin and eosin staining. Despite the long interval of prednisolone administration, lymphocyte infiltration was recognized in our patient. Immunostaining revealed predominantly CD8^+^ lymphocytes, whereas few CD4^+^ lymphocytes were detected. These findings suggested that this hepatic irAE was an acute response. To our knowledge, this patient’s case is the first of steroid-refractory hepatic irAE that was successfully treated with UDCA and bezafibrate, although it has been reported that patients with grade 1 or 2 hepatic irAEs involving bile duct disorders improved with administration of UDCA [15].

In general, UDCA is expected to improve cholestasis by biliary secretion and to alleviate the hepatocellular injury by replacing cytotoxic hydrophobic bile acids. Moreover, UDCA has recently been shown to be effective for PBC, a condition mediated (in part) by immunosuppression [19]. On the other hand, bezafibrate is a fibric acid derivative commonly used in the management of lipid disorders. The combination of UDCA and bezafibrate is thought to be effective in patients with PBC [12]. The proposed mechanism of action of fibric acid derivatives in PBC involves regulating the production of various kinds of lipids and proteins through the activation of peroxisome proliferator-activated receptor-α (PPARα). Activated PPARα in turn can inhibit nuclear factor-κB activation, decreasing the immune response [20]. In Japan, there are no indications for bezafibrate other than hyperlipidemia, so sufficient informed consent is required for use of this medication. It is possible that these two drugs (UDCA and bezafibrate) synergize, leading to the results seen in our patient’s case.

Immune checkpoint inhibitors are widely used to treat various types of cancer. Careful use of irAEs thus is important because subsequent cancer therapy may be markedly impacted.

## Conclusion

In cases of steroid-refractory immune-related hepatitis, the combination of UDCA and bezafibrate should be considered before using conventional strong immunosuppressants.

## Data Availability

Data are available from the corresponding author on reasonable request.

## Abbreviations

ALP: Alkaline phosphatase
ALT: Alanine aminotransferase
AST: Aspartate aminotransferase
CMV: Cytomegalovirus
CTLA-4: Cytotoxic T-lymphocyte-associated antigen 4
EBV: Epstein-Barr virus
γ-GTP: γ-Glutamyltranspeptidase
IgG: Immunoglobulin G
IgM: Immunoglobulin M
irAE: Immune-related adverse events
MMF: Mycophenolate mofetil
PBC: Primary biliary cirrhosis
PD-1: Programmed cell death 1
PD-L1: Programmed death ligand 1
PPARα: Peroxisome proliferator-activated receptorα
UDCA: Ursodeoxycholic acid
VCA: Virus capsid antigen
